# Genotypic Distribution and Epidemiological Analysis of Hepatitis C Virus in the Epirus Region of Northwestern Greece (2014–2024)

**DOI:** 10.3390/diseases14040126

**Published:** 2026-04-01

**Authors:** Petros Bozidis, Christos Kittas, Alexandra Myari, Konstantinos Patras, Konstantina Gartzonika

**Affiliations:** 1 Department of Microbiology, Faculty of Medicine, School of Health Sciences, University of Ioannina, 45110 Ioannina, Greece; alekamiari@yahoo.com (A.M.); kgartzon@uoi.gr (K.G.); 2 Department of Microbiology, University Hospital of Ioannina, 45500 Ioannina, Greece; ckittas@gmail.com (C.K.); kwn.patras@gmail.com (K.P.)

**Keywords:** hepatitis C virus, HCV genotype, epidemiology, Greece, viral load, age, gender

## Abstract

Background/Objectives: This retrospective study investigates the prevalence and distribution of HCV genotypes among 233 genotyped patients from the Epirus region of Northwestern Greece from 2014 to 2024. Methods: Genotypes were detected by molecular diagnostic assays, and their association with demographic parameters and viral load was analyzed. Results: The most prevalent subtype was 3a (50.2%), especially among younger and male patients, followed by subtypes 1b and 1a. A statistically significant association was found between genotype and both age and sex, while genotype distribution did not significantly differ by national origin. Furthermore, subtype 6c-I was found only in a non-native case, suggesting a possible introduction of this rare strain. Viral load showed no significant difference by sex, genotype, or age group. A notable decline in HCV cases was documented during the COVID-19 pandemic, underscoring the impact of the public health crisis on HCV diagnosis. Despite the decreasing need for genotyping in the direct-acting antiviral (DAA) era, our findings support the continued molecular surveillance of circulating HCV strains. Conclusions: This is the first study to longitudinally assess HCV genotype dynamics over a full decade (2014–2024) in the Epirus region of Northwestern Greece, capturing trends during the COVID-19 era and documenting the emergence of rare genotypes. It contributes to the evolving knowledge of HCV epidemiology in Southeastern Europe.

## 1. Introduction

Hepatitis C virus (HCV) infection remains a significant global health concern, with an estimated 50 million people chronically infected and 1 million new cases occurring annually [1]. In addition, HCV was responsible for approximately 221,000 deaths in 2022, most of which were attributed to cirrhosis and hepatocellular carcinoma [2]. These figures are largely explained by the asymptomatic nature of the infection, its potential for severe long-term consequences, and the substantial proportion of undiagnosed or untreated HCV carriers, particularly in resource-limited settings [2,3].

The global epidemiology of HCV is characterized by considerable variation across different regions and populations in terms of prevalence, transmission patterns, and genotype distribution [4,5]. In low- and middle-income countries—particularly in North and Sub-Saharan Africa, as well as in Central and East Asia—HCV prevalence appears still high, mainly due to healthcare practices such as unsafe medical injections and limited access to screening services [4]. In contrast, high-income countries report lower HCV prevalence in the general population but an increasing burden among specific high-risk groups—primarily people who inject drugs (PWID), who account for up to 79% of new infections [6]. In European Union countries (EU/EEA), 1.8 million people (0.5%) were estimated to be infected in 2022, although substantial heterogeneity existed across countries [7]. Variations in genotype distribution and predominant transmission modes across Northern, Western, and Southern Europe are shaped by region-specific public health histories and the demographic composition of affected high-risk groups [8].

In Greece, epidemiological data on HCV remain relatively limited, especially regarding integrated analyses that incorporate demographic variables, clinical context and viral load [9]. According to the available reports, genotype 1—particularly subtype 1b—was historically predominant, especially among older individuals with a history of blood transfusion before 1992 or exposure to invasive medical procedures [10,11]. The progressive implementation of rigorous blood screening protocols contributed to a significant reduction in transfusion-related HCV infections, which in turn led to changes in genotype distribution characterized by an increasing representation of genotype 3 infections, largely driven by transmission among people who inject drugs (PWID) [10,11]. This trend reflects broader changes in HCV transmission dynamics, with genotype 3 becoming increasingly prevalent among younger male PWID, a group that continues to exhibit high infection rates despite recent declines [10,12]. Therefore, continued monitoring, targeted detection and genotype determination remain essential for understanding current patterns of transmission, updating control strategies, and characterizing patients’ risk for therapeutic failure.

This study aims to investigate the distribution of HCV genotypes among patients diagnosed in Epirus (Northwestern Greece) over a decade (2014–2024). Epirus is a distinct administrative region characterized by long-standing cross-border mobility with the Western Balkans, particularly Albania, as well as more recent refugee and migrant flows from the Middle East and South Asia [13,14]. Through a comprehensive retrospective analysis conducted for the first time in this region, we aimed to discern patterns that may inform future prevention, screening, and treatment strategies at both regional and national levels. Although the introduction of pan-genotypic direct-acting antiviral agents (DAAs) has reduced the dependence of treatment decisions on genotype, HCV genotyping may still retain clinical value in specific scenarios, including cirrhosis assessment, treatment optimization and the identification of patients at risk for therapeutic failure or relapse [15,16]. In addition, the efficacy of DAAs may be influenced by the presence of rare or unusual HCV genotypes that are underrepresented in clinical trials conducted in industrialized countries and are more frequently reported in Africa and Asia [17,18]. This consideration becomes particularly relevant in regions experiencing increased population mobility and migration, where the circulation of less common HCV genotypes may be observed. In this context, long-term regional datasets can provide valuable information that may not be captured by national surveillance systems. Therefore, the primary aim of this study was to describe the distribution of HCV genotypes and their associations with demographic variables (age, sex, nationality). Secondary analyses also explored potential differences across clinical setting, and viral load measurements.

## 2. Materials and Methods

### 2.1. Study Design and Population

This retrospective study included 236 serum samples obtained from 235 individuals with confirmed HCV infection, either newly diagnosed or with established chronic hepatitis C, who were referred to our laboratory for HCV genotyping and viral load analysis between July 2014 and June 2024. Of these, 233 samples were successfully genotyped. Samples from individuals under follow-up for chronic hepatitis C who underwent repeat HCV genotyping were not re-included in the study if the genotype was identical to that of a previous analysis, as such cases did not represent new infection or distinct viral strains. For genotype distribution and statistical analyses, each individual was included only once, based on the first available genotyping result. In the single case where a different HCV genotype was detected at a later time point, this finding was interpreted as a reinfection event and was reported descriptively but was not included as a separate case in the primary analysis. No other exclusion criteria were applied, apart from insufficient sample volume or failed amplification.

All specimens were analyzed at the Microbiology Laboratory of the University General Hospital of Ioannina (UGHI), an 845-bed tertiary care center located in the city of Ioannina, the regional capital. Most samples originated from UGHI, with a smaller number referred from secondary hospitals and healthcare facilities across Epirus. Demographic characteristics (age, sex, nationality), clinical origin of each sample, and laboratory data (viral load, genotype) were retrieved from laboratory records in anonymized form and used exclusively for research purposes.

### 2.2. HCV RNA Quantification

RNA was extracted from 200 μL of serum using the High Pure Viral Nucleic Acid Kit (Roche Diagnostics GmbH, Mannheim, Germany), according to the manufacturer’s instructions. Quantification of HCV RNA was carried out using the cobas^®^ HCV assay in combination with the cobas^®^ 4800 system (Roche Molecular Diagnostics, Pleasanton, CA, USA), an automated, real-time reverse transcription polymerase chain reaction (RT-PCR) platform targeting conserved regions of the HCV genome. The dynamic range of the assay is 15 to 10^8^ international units per milliliter (IU/mL), with the limit of detection (LOD) of 7.6 IU/mL in serum and a lower limit of quantification (LLOQ) of 15 IU/mL [19]. All results were expressed in IU/mL. A total of 235 RNA quantification measurements were performed, corresponding to one per patient, except for a single case in which quantification was repeated due to confirmed reinfection after a 25-month interval.

### 2.3. HCV Genotyping

Genotype determination was performed using the VERSANT^®^ HCV Genotype 2.0 Assay (Siemens Healthcare GmbH, Erlangen, Germany), which remains widely used in routine diagnostic settings despite the availability of newer technologies [20,21]. This line-probe assay (LiPA) is a reverse-hybridization-based test that detects HCV genotypes by hybridizing amplified, biotinylated PCR products to genotype-specific oligonucleotide probes immobilized on nitrocellulose strips. The RT-PCR primers used in this assay target both the 5′ untranslated region (5′UTR) and the core region of the HCV genome, enabling not only detection but also improved and more precise discrimination of genotypes 1 to 6, including subtypes 1a and 1b, as well as a broad range of subtypes within genotype 6 (e.g., 6a, 6d, 6e, 6f, 6i, 6l and 6n) [20]. Interpretation of genotyping results was conducted according to the manufacturer’s instructions. Samples with weak or ambiguous hybridization signals, most likely related to low viral load, were excluded from subtype-specific analyses. However, the overall HCV genotype could still be determined based on conserved genomic regions targeted by the assay; therefore, these samples were classified as non-subtypeable genotypes (genotype 1 or genotype 3) and were included in genotype-level analyses.

### 2.4. Ethical Considerations

All data were anonymized prior to analysis. The study was conducted in accordance with applicable guidelines and regulations and was approved by the relevant Institutional Review Board. Due to the retrospective nature of the study and the use of anonymized laboratory data collected as part of routine diagnostic procedures, the requirement for informed consent was waived. No patient-identifiable information was accessed or analyzed.

### 2.5. Statistical Analysis

Descriptive statistics were performed on the total genotyped cohort (n = 233). In addition, temporal trends in genotype distribution were explored descriptively. No formal statistical trend analysis was performed, as the number of cases per six-month interval was limited and uneven—particularly for less frequent genotypes. However, to ensure sufficient statistical power and avoid sparse data cells in multivariable modeling, comparative analyses were restricted to the three most prevalent subtypes: 3a, 1a, and 1b (n = 194). Subtypes with low frequency (<10%) and non-subtypeable samples were excluded from the inferential models to prevent instability in parameter estimation. Continuous variables such as age and viral load were expressed as medians and interquartile ranges (IQR), while categorical variables were presented as frequencies and percentages. For age-stratified analyses, patients were categorized into two groups (<50 and ≥50). This cut-off was selected as an epidemiologically meaningful proxy to distinguish younger individuals more likely to reflect recent transmission patterns—predominantly associated with injection drug use—from older cohorts historically infected through blood transfusions or medical procedures prior to the implementation of systematic HCV screening. Such age thresholds or age-based cohort stratifications have been commonly used in similar epidemiological studies of HCV genotype previously [10,11].

Differences in age across HCV subtypes were analyzed using the Kruskal–Wallis test, followed by Dunn’s post hoc test with Bonferroni correction for pairwise comparisons. Associations between subtype and categorical variables (gender and nationality) were assessed using Fisher’s exact test. Differences in viral load across HCV subtypes were analyzed using the Kruskal–Wallis test, followed by Dunn’s post hoc test with Bonferroni correction for pairwise comparisons, while differences in viral load across age categories and gender were analyzed using Mann–Whitney U test.

To identify factors associated with HCV subtype (1a, 1b, or 3a), multinomial logistic regression models were constructed using the vglm function (Vector Generalized Linear Models) from the VGAM R package (version 1.1-5). The response variable was the HCV subtype, with subtype 1b serving as the reference category. Predictor variables included standardized age (Zage), gender, and nationality. Three nested models were evaluated to determine the most parsimonious fit for the data: a baseline additive model that assessed the independent main effects of age, gender, and nationality; a model that included an interaction term between age and gender to determine if age-related subtype trends differed by gender and a model that included an interaction term between age and nationality to evaluate ethnic variations in age-subtype associations. Model comparison was performed using the Likelihood Ratio Test (LRT) and the Akaike Information Criterion (AIC). Model fit was assessed using McFadden’s Pseudo-R2, regression results are reported as adjusted odds ratios (OR) with 95% confidence intervals (CI) and population-level effects were visualized using predicted probability distributions with 95% confidence intervals calculated via the Delta method. A *p*-value < 0.05 was considered statistically significant. All analyses were performed using the R statistical environment (version 4.3.1). Temporal trends in genotype distribution were explored descriptively. No formal statistical trend analysis was performed, as the number of cases per six-month interval was limited and uneven-particularly for less frequent genotypes.

## 3. Results

### 3.1. Study Population, Genotype Detection and Temporal Trends in HCV Distribution

**Figure 1:**
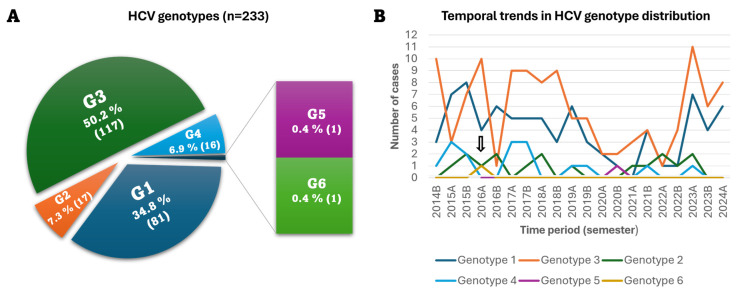
HCV genotype distribution in the study population. (**A**) Percentage and absolute number of patients infected with each HCV genotype. (**B**) Temporal trends in genotype distribution across six-month intervals from 2014B (B: second half of the year) to 2024A (A: first half of the year). This panel provides a descriptive temporal overview; no formal statistical trend analysis was performed. The arrow indicates the introduction of a novel genotype (6c–i) potentially related to immigration.

The study analyzed specimens from 235 patients collected over a ten-year period (2014–2024), with ages ranging from 20 to 85 years. Of these, 233 samples were successfully genotyped. The median age was 44 years (interquartile range [IQR]: 35.5–55), with 147 individuals (63.52%) being under 50 years of age and 86 (36.91%) aged 50 years or older. The majority of patients were male (75.11%, n = 175), whereas 24.89% were female (n = 58). Most samples were derived from the Outpatient department (n = 135), followed by the Internal Medicine (n = 54) and the Gastroenterology wards (n = 13) (see Supplementary Table S1). Genotype 3 was the most prevalent in the cohort, followed by genotype 1 (Figure 1A). It should be noted that in four cases, (three for genotype 1 and one for genotype 3), subtype assignment was not possible, most likely due to weak or ambiguous hybridization signals. Genotype 2, including subtypes 2a/2c and 2b, was detected in 7.3% of cases, and genotype 4 in 6.9%, while genotypes 5 and 6 were rare (0.4% each). Notably, in one of the patients, a different genotype (a shift from 2a/2c to 3a) was detected within a 25-month interval. While this may represent a case of reinfection, other possibilities—such as superinfection or technical error—cannot be excluded. Such cases of reinfection with a distinct HCV strain have been documented in the literature [22]. This case was analyzed descriptively and was not included as a separate observation in the genotype distribution analysis.

To investigate temporal trends in genotype prevalence, HCV genotypes were plotted across consecutive six-month intervals between 2014B and 2024A (‘where “A” denotes the first half of the year [January–June] and “B” the second half [July–December]). As shown in Figure 1B, genotype 3 was the most frequently observed strain across most time intervals, though no formal trend analysis was applied. Genotype 1 also exhibited a fluctuating pattern in line with the overall detection of HCV in the Epirus region of Northwestern Greece. Genotypes 2, 4, 5 and 6 remained rare, with sporadic detection and no clear trend over time.

### 3.2. HCV Subtype Distribution by Age, Sex, and Nationality

**Figure 2:**
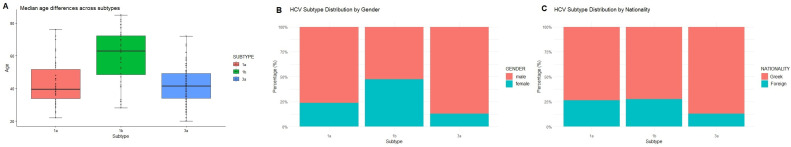
Distribution of the three most prevalent HCV subtypes (1a, 1b and 3a) according to age (**A**), gender (**B**) and nationality (**C**).

Median age differed significantly across HCV subtypes (Kruskal–Wallis *x*^2^ = 52.4, *p* < 0.001). Post hoc pairwise comparisons using Dunn’s test with Bonferroni correction indicated that patients with subtype 1b [63.0 years (25.3)] were significantly older than those with subtypes 1a [39.5 years (20.5), *p* < 0.001] and 3a [41.5 years (15.8), *p* < 0.001], whereas no significant age difference was observed between subtypes 1a and 3a (*p* = 1.00) (Figure 2A).

Fisher’s exact test revealed significant associations between HCV subtype and both gender (*p* < 0.001) and nationality (*p* = 0.036). Specifically, subtype 3a predominated among male patients, whereas subtype 1b showed a higher proportional representation among females (47.5%) compared to subtypes 1a (23.7%) and 3a (12.9%) (Figure 2B). Similarly, the distribution of subtypes varied significantly by nationality, with 3a being the most frequent across both groups but showing a distinct clustering pattern (Figure 2C).

### 3.3. Viral Load, Genotype, Age and Gender

**Figure 3:**
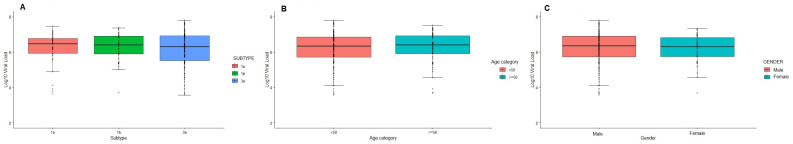
Viral load analysis in relation to subtype and demographic factors. (**A**) Boxplots of HCV viral load (log_10_ IU/mL) across subtypes. (**B**) Boxplots of viral load (log_10_ IU/mL) across patient age-category. (**C**) Boxplots of viral load (log_10_ IU/mL) according to patient gender.

There was no evidence that median viral load differed significantly across HCV subtypes (Kruskal–Wallis *x*^2^ = 0.18, *p* = 0.91) (Figure 3A). Similarly, there was no evidence that median viral load differed significantly between patients < 50 y vs. patients ≥ 50 y (W = 4067, *p*-value = 0.56) (Figure 3B). Finally, there were no evidence that median viral load differed between male patients vs. female patients (W = 3408.5, *p* = 0.62) (Figure 3C).

### 3.4. Multinomial Regression Analysis of HCV Infection Predictors

Preliminary model comparisons using Likelihood Ratio Tests (LRT) indicated that the inclusion of interaction terms between age and gender (*x*^2^ = 3.06, *p* = 0.22) or age and nationality (*x*^2^ = 5.08, *p* = 0.08) did not significantly improve model fit. Therefore, the baseline additive model (main effects only) was selected for the analysis.

The baseline additive model demonstrated an overall good fit to the data, with a McFadden’s pseudo-R^2^ of 0.18, suggesting that the combination of age, gender, and nationality provides a meaningful explanation for the observed distribution of HCV subtypes 1a, 1b, and 3a.

The multivariate multinomial logistic regression (using subtype 3a as the reference) revealed that age was a powerful predictor for subtype 1b. Specifically, an increase of one standard deviation in age was associated with a 3.86-fold increase in the odds of presenting with subtype 1b (95% CI: 2.38–6.26; *p* < 0.001). Furthermore, gender was significantly associated with subtype 1b, with females having 4.24 times higher odds than males of being infected with 1b rather than 3a (95% CI: 1.57–11.41; *p* = 0.004). In contrast, no significant differences in age (OR: 1.20, 95% CI: 0.77–1.86; *p* = 0.417) or gender (OR: 2.00, 95% CI: 0.78–5.11; *p* = 0.148) were found when comparing subtype 1a to 3a, suggesting these two variants circulate within similar demographic groups.

**Figure 4:**
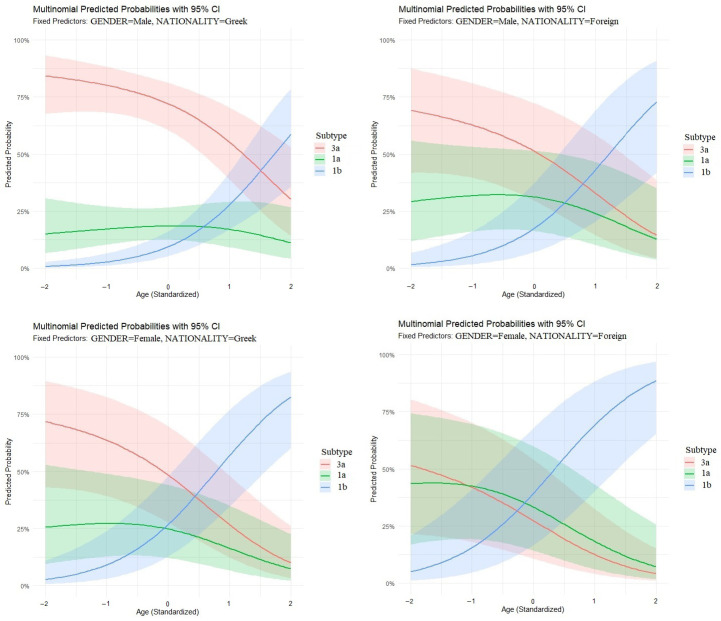
Multivariate multinomial regression analysis of factors associated with HCV genotype distribution. Predicted probabilities of HCV genotypes (3a, 1a, and 1b) across standardized age values (Z-scores), derived from an additive (main effects only) multinomial logistic regression model at various combinations of fixed gender and nationality.

Predicted probability plots (Figure 4) demonstrated a consistent trend toward an age-related increase in subtype 1b across all demographic strata. However, the precision of these estimates varied by subgroup; while the model showed high narrow-interval confidence for the majority cohort (males, Greek) (Figure 4, upper left panel), wider 95% confidence intervals were observed for females and Nationality 2, reflecting the smaller sample sizes in these specific subpopulations.

## 4. Discussion

This study provides the first longitudinal analysis of HCV genotype distribution in the Epirus region of Northwestern Greece over a ten-year period (2014–2024), examining viral load patterns and demographic characteristics in a population shaped by distinct demographic dynamics and healthcare structures. The findings contribute to a better understanding of the regional transmission dynamics, which remain insufficiently characterized in this part of the country. Subtype 3a was the most frequently identified strain, while subtypes 1b and 1a followed in prevalence. Together, these three subtypes have shaped the regional HCV epidemiology and remained the dominant variants throughout the study period. These findings are not unique to our region. Similar trends have been reported across Europe, with an important distinction: in most European countries, genotype 1 remains predominant, followed by genotype 3 [23,24]. However, recent studies from Greece are consistent with our results, indicating an increasing representation of genotype 3 infections in recent years [25,26]. This pattern may be partly attributed to changes in transmission routes, as well as to the availability, accessibility, and genotype-specific efficacy of recommended antiviral treatments over the past two decades [25,26]. Notably, during the study period, a marked decline in newly reported HCV cases was recorded between late 2019 and mid-2022. This interval coincides with the emergence and subsequent waves of the COVID-19 pandemic in the region of Epirus, as thoroughly documented elsewhere [27]. The implementation of strict public health measures to contain SARS-CoV-2—such as lockdowns, travel restrictions, and the prioritization of acute COVID-19 care—likely limited healthcare access for people with chronic HCV infection. As a result, reduced HCV testing and underreporting of new cases during this period may explain the observed decline, an effect that has also been documented across several European countries and globally [28,29,30]. However, this interpretation remains speculative, as direct data on healthcare utilization and HCV testing rates during the pandemic period were not available.

Our data also revealed a significant association between age and the three most common HCV subtypes (Kruskal–Wallis test, *p* < 0.001), with subtypes 3a and 1a showing lower median age compared to subtype 1b (Figure 2A). These subtypes were especially prevalent among younger individuals, particularly those under 50 years of age. Furthermore, multinomial regression analysis showed that each standard deviation increase in age was associated with a 3.86-fold increase in the odds of infection with subtype 1b relative to subtype 3a. Other studies around the world consistently confirm this higher prevalence of both subtype 3a and subtype 1a among individuals under 50 years of age [31,32]. Subtype 3a is strongly associated with intravenous drug use (IVDU), which is more common among younger populations [33,34]. The global spread of this genotype, which originated from South Asia, has been driven by migration to Europe and other high-income regions [35]. On the other hand, subtype 1a, which is also frequently associated with IVDU but less so with migration, is more established in high-income countries, where genotype 1 has traditionally predominated due to historical blood product contamination [36]. While transmission-related factors provide the most plausible explanation for the predominance of subtypes 3a and 1a among younger individuals, the potential contribution of genotype-specific biological characteristics has also been discussed. Although such distinct biological features have not been clearly described in the literature for subtype 1a, subtype 3a has been shown in experimental studies to exhibit unique replication dynamics, including enhanced lipid droplet association and a more pronounced steatogenic effect on hepatocytes, both of which are believed to contribute to the efficiency of viral replication complexes [37,38]. Based on these observations, it has been hypothesized that subtype 3a may potentially exhibit enhanced replication efficiency in hosts with higher metabolic activity typically found in younger individuals, particularly during early stages of infection [39,40]. Such an interplay could theoretically result in higher viral titers. However, in our cohort, no statistically significant differences in viral load were observed among HCV subtypes, suggesting that genotype-specific biological properties did not translate into measurable differences in circulating HCV RNA levels. In conclusion, our results indicate that age-subtype associations are primarily driven b transmission dynamics and epidemiological factors, while the role of genotype-specific biological characteristics remains an open question warranting further investigation. As far as subtype 1b is concerned, it was more frequently detected among individuals over 50 years of age. These findings, particularly for subtype 1b, are consistent with previously published data on HCV epidemiology in Greece and other European countries [25,26].

A statistically significant association was identified between gender and the distribution of HCV subtypes, although the male-to-female ratio within our study population was not balanced (75% male patients). Recognizing that this gender imbalance may limit the power of sex-stratified analyses, particularly for less common subtypes, the following patterns were observed. Subtype 3a was more frequently detected among male patients and was estimated as more likely (66.9%) than either 1a (19.2%) or 1b (13.9%) by multinomial regression. In contrast, the odds of subtype 1b relative to 3a were approximately fourfold higher in female patients than in male patients. In addition, several less common genotypes (e.g., genotypes 1, 2, 3, and subtype 6c–l) were detected exclusively in male patients, while genotype 5a was identified only in a single female patient. The latter is one of the rarest genotypes detected in Greece [26]. Previous studies have also reported associations between specific genotypes and gender [41,42]. However, such associations may vary across geographic regions and are likely shaped by gender-linked behaviors, differential exposure risks, and broader sociocultural factors [43]. Beyond these epidemiological determinants, experimental and clinical evidence suggests that biological sex-related differences, including the immunomodulatory effects of sex hormones, may influence host immune responses and interferon signaling. Experimental data indicate potential effects on HCV replication dynamics, whereas clinical studies have mainly linked sex-related differences to disease course and treatment outcomes [44,45,46,47]. Still, supporting evidence remains limited, highlighting the need for further research to clarify the role of biological sex in shaping genotype-specific HCV patterns. Interestingly, in contrast to the observed associations between genotype and gender, viral load did not significantly differ between male and female patients for any of the genotypes examined.

One of the most noteworthy findings of our study was the striking similarity in HCV genotype distribution between patients of non-Greek origin and those of Greek nationality. Greece has been a major destination for intense migration flows over the past thirty years, which significantly increased after 2015 [13,14]. Early migration primarily involved neighboring Balkan countries during the 1990s through 2010, while more recent waves have originated from Middle Eastern and African countries, potentially contributing to the introduction of novel pathogens into the national population [13,14]. In the context of our study, and due to Greece’s long-standing migration history, it is plausible that a number of these individuals have been permanent residents for decades and may have acquired the infection locally. Unfortunately, such information was not available. Although patients of non-Greek origin did not significantly alter the overall HCV genotype landscape in our region, one noteworthy observation was the identification of genotype 6c-I in a patient of non-Greek origin. To the best of our knowledge, this subtype has not been previously reported in Greece. While this finding may suggest the introduction of a rare genotype via migration, alternative explanations, such as undocumented local transmission, cannot be ruled out. Importantly, as this observation is based on a single case, its epidemiological significance should be interpreted with caution. Moreover, because the identification was based on a line-probe assay and only a single case was available, potential technical limitations of the assay, including misclassification or laboratory artifact, cannot be excluded. Therefore, further investigation, including viral genome sequencing and phylogenetic analysis, would be required to clarify its origin.

A limitation of this study is that additional clinical and behavioral information such as history of intravenous drug use, history of blood transfusion, time of residence in the country for non-native patients, alcohol consumption, comorbidities like chronic kidney disease, body mass index (BMI) and other clinical variables were not available. Although the core epidemiological and virological parameters (age, sex, nationality, genotype, and viral load) were sufficient to capture the epidemiological picture of the region, the analysis would have been more complete if such parameters had been incorporated into our study. This highlights the need for future prospective studies where such variables will be actively collected and integrated with molecular data. Additionally, since all samples originated from a tertiary care center and no primary care or community-based data were included, this may introduce a degree of selection bias. We acknowledge this as a limitation of the current study design, and it should be considered when interpreting genotype distributions.

## 5. Conclusions

In the era of highly effective pan-genotypic DAAs, the need for routine HCV genotyping prior to treatment initiation has markedly declined [48]. Nevertheless, continued molecular epidemiological surveillance remains important for monitoring circulating genotypes and detecting uncommon variants that may emerge through population mobility and migration, as well as in selected clinical scenarios [15,16,48]. In this context, the present study provides, for the first time, a longitudinal and detailed mapping of circulating HCV strains in the Epirus region of Northwestern Greece over a ten-year period. These findings contribute new regional data to the evolving epidemiological landscape of HCV in Southeastern Europe. From a public health perspective, continued investment in laboratory-based molecular epidemiological surveillance remains an integral component of national HCV elimination strategies, complementing simplified diagnostic pathways and supporting targeted prevention and control efforts.

## Data Availability

The data presented in this study are available in Supplementary Table S1. All patient-identifying information has been removed to ensure anonymity. Additional individual-level data is available from the corresponding author upon reasonable request but is not publicly available due to privacy and ethical considerations.

## Supplementary Materials

The following supporting information can be downloaded at https://www.mdpi.com/article/10.3390/diseases14040126/s1: Supplementary Table S1: Demographic, clinical and virological characteristics of HCV-infected patients in the study (2014–2024).

## Abbreviations

The following abbreviations are used in this manuscript:

HCV	Hepatitis C virus
DAAs	Direct-acting antiviral agents
PWID	People who inject drugs
RT-PCR	Reverse transcription polymerase chain reaction
LOD	Limit of detection
LLOQ	Lower limit of quantification
IQR	Interquartile range
IVDU	Intravenous drug use
COVID-19	Coronavirus disease caused by SARS-CoV-2
